# Supplementary material for: Nampt-mediated spindle sizing secures a post-anaphase increase in spindle speed required for extreme asymmetry
Source: Nat Commun. 2020 Jul 7;11:3393. doi: 10.1038/s41467-020-17088-6 (PMC7341875; doi:10.1038/s41467-020-17088-6)
Supplement: Supplementary file 1 — Supplementary Information [file 41467_2020_17088_MOESM1_ESM.pdf]

Supplementary Information

Nampt-mediated spindle sizing secures a post-anaphase increase in spindle speed required for extreme asymmetry

Wei et al.

Title: Nampt-mediated spindle sizing secures a post-anaphase increase in spindle speed required for extreme asymmetry

Authors: Zhe Wei[1], Jessica Greaney[1], Wei-Guo Nicholas Loh[1] and Hayden Homer[1,*]

Affiliation:

[1]The Christopher Chen Oocyte Biology Research Laboratory, Centre for Clinical Research, The University of Queensland, Herston, QLD 4029, Australia.

***Corresponding Author:** Professor Hayden Homer

*Correspondence to: h.homer@uq.edu.au

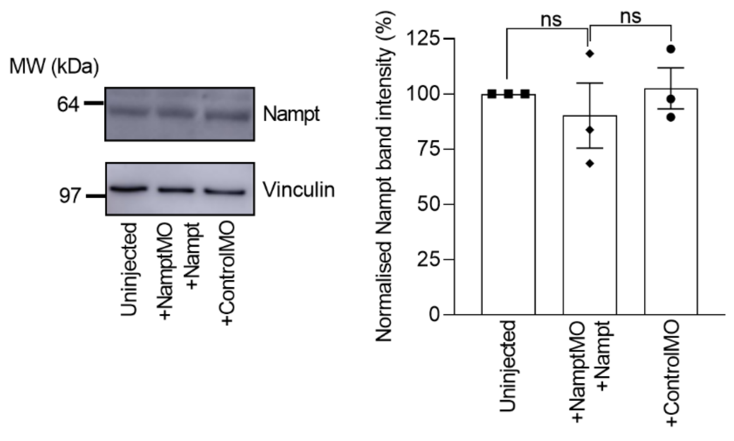

Supplementary Figure 1.

Nampt levels following recombinant Nampt injection in Nampt-depleted oocytes. Uninjected, NamptMO+Nampt- and ControlMO-injected oocytes were immunoblotted for Nampt. Vinculin served as a loading control (left); n=20 oocytes per lane. Western blots were repeated three times. Quantification of Nampt protein levels (right). Data in graph are presented as the mean ± SEM and each data point represents the normalised band intensity in each Western blot. *P* values, ns denoted $P > 0.05$. Statistical comparisons were made using a one-way ANOVA. Source data are provided as a Source Data file.

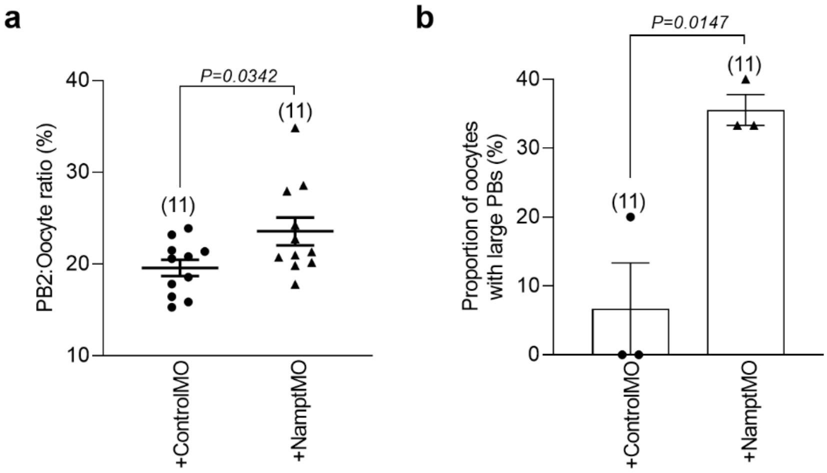

Supplementary Figure 2.

PB2 size is increased following Nampt depletion. (**a**) Ratio of PB2 height to oocyte diameter. (**b**) Proportion of oocytes with large PB2s. See Methods for further details. Oocyte numbers are shown in parenthesis from three independent experiments and each data point represents oocyte proportions. Data in graphs are presented as the mean ± SEM. *P* values are shown in the graphs. Statistical comparisons were made using two-tailed Student's *t*-test in (a and b). Source data are provided as a Source Data file.

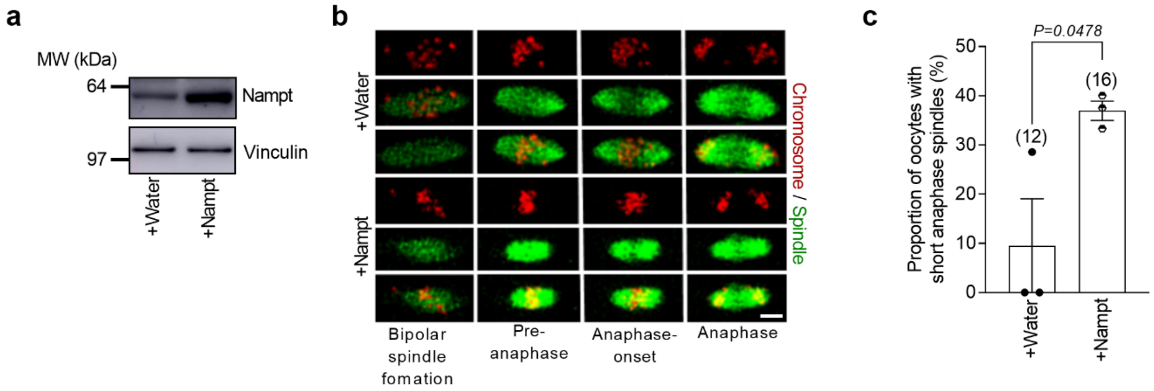

Supplementary Figure 3.

Overexpression of Nampt produces shorter spindles. (**a**) Water- and recombinant Nampt-injected oocytes were immunoblotted for Nampt. Vinculin served as a loading control; n=15 oocytes per lane. A representative blot from three independent experiments is shown. (**b**) Shown are panels of images comprised of selected fluorescence frames from representative timelapse series of spindles labelled with SiR-tubulin and chromosomes expressing H2B-RFP. Scale bar, 10µm. Representative images from three independent experiments are shown. (**c**) Proportion of oocytes with short anaphase spindles. Anaphase spindle length was measured at 10 min post-anaphase-onset. See Methods for further details. Oocyte numbers are shown in parenthesis from three independent experiments (c). Data in graphs are presented as the mean ± SEM and each data point represents oocyte proportions. *P* values are shown in the graphs. Statistical comparison was made using a two-tailed Student's *t*-test in (c). Source data are provided as a Source Data file.

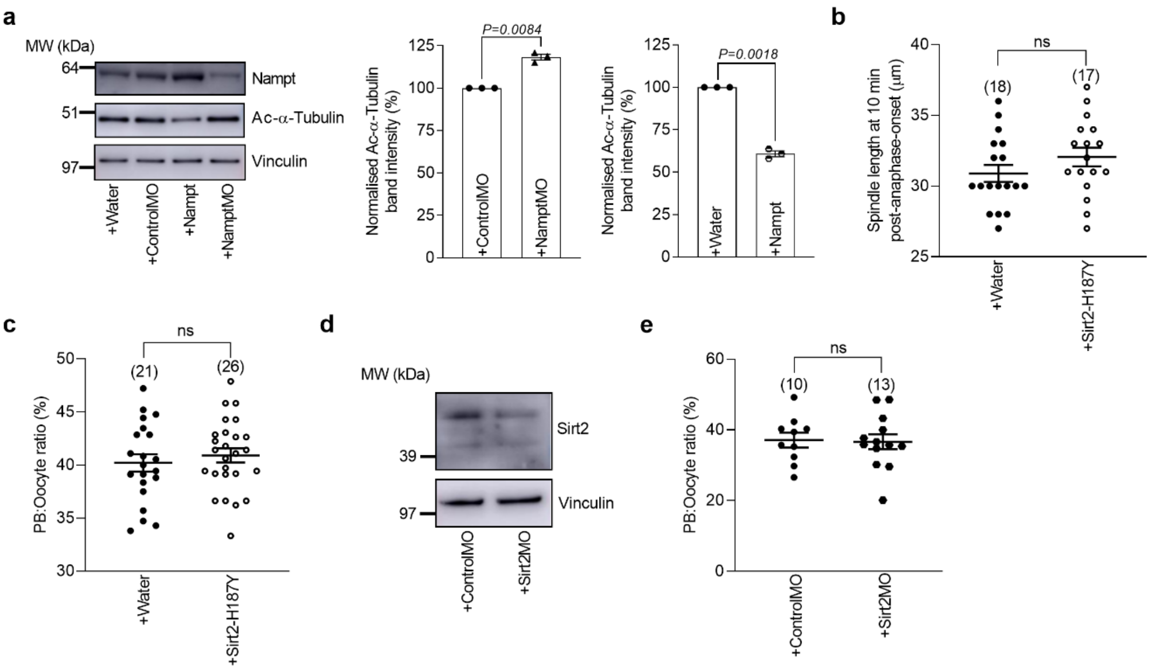

Supplementary Figure 4.

Neither Sirt2-H187Y nor Sirt2 knockdown compromises asymmetry or spindle size. (**a**) Water-, controlMO-, recombinant Nampt- and NamptMO-injected oocytes were immunoblotted for Nampt and acetylated-α-Tubulin (Ac-α-Tubulin). Vinculin served as a loading control (left); n=15 oocytes per lane. Western blots were repeated 3 times. Band intensities of Ac-α-Tubulin were quantified and compared as shown (right) with each data point in graphs representing the normalised band intensity in each Western blot. (**b**) Lengths of spindles at 10 min post-anaphase-onset in water- and Sirt2-H187Y-injected oocytes. (**c**) Ratio of PB width to oocyte diameter in water- and Sirt2-H187Y-injected oocytes. (**d**) ControlMO- and Sirt2MO-injected oocytes were immunoblotted for Sirt2. Vinculin served as a loading control; n=29 oocytes per lane. A representative blot from three independent experiments is shown. (**e**) Ratio of PB width to oocyte diameter in ControlMO- and Sirt2MO-injected oocytes. Oocyte numbers are shown in parenthesis from three independent experiments (b, c and e). Data in graphs are presented as the mean ± SEM. *P* values are shown in the graphs, ns denoted $P > 0.05$. Statistical comparisons were made using two-tailed Student's *t*-test in (a, b, c and e). Source data are provided as a Source Data file.

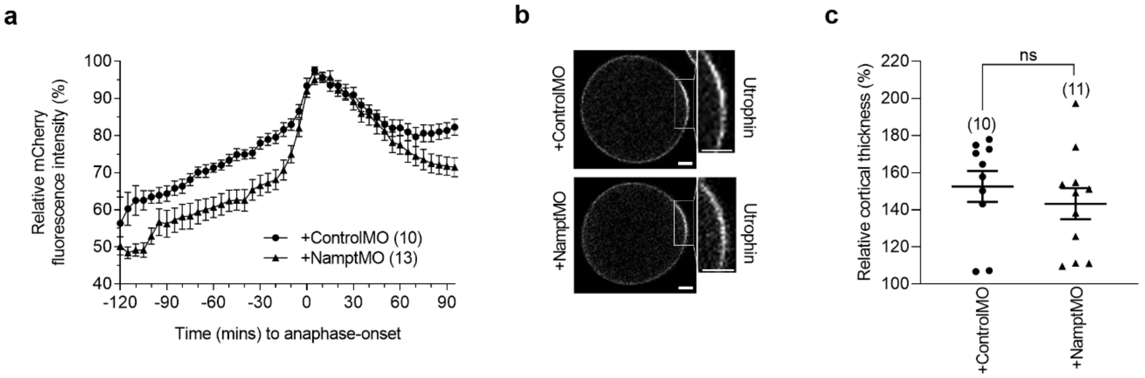

Supplementary Figure 5.

Nampt-depletion does not affect cytoplasmic or cortical F-actin during the peri-anaphase period. (**a**) Cytoplasmic UtrCH-mCherry intensity in ControlMO- or NamptMO-injected oocytes relative to anaphase-onset. Fluorescence intensity was normalised to its highest expression in each oocyte. Note that cytoplasmic F-actin intensity increased to similar extents coincident with anaphase-onset in both groups. (**b**) Shown are images of representative mock- and Nampt-depleted oocytes expressing UtrCH-mCherry at 5 min prior to anaphase-onset. Scale bars, 10μm. Representative data from three independent experiments are shown. (**c**) The graph shows relative cortical F-actin thickness at 5 min prior to anaphase-onset. See Methods for further details. Oocyte numbers are shown in parenthesis from three independent experiments. Data in graphs are presented as the mean ± SEM. P value, ns denoted $P > 0.05$. Statistical comparison was made using a two-tailed Student's t-test. Source data are provided as a Source Data file.

Supplementary Table 1. Primer used in this study

Primer name	Primer sequence 5’-3’
T3	GCAATTAACCCTCACTAAAGG
