# Supplementary material for: Nampt-mediated spindle sizing secures a post-anaphase increase in spindle speed required for extreme asymmetry
Source: Nat Commun. 2020 Jul 7;11:3393. doi: 10.1038/s41467-020-17088-6 (PMC7341875; doi:10.1038/s41467-020-17088-6)
Supplement: Supplementary file 3 — Description of Additional Supplementary Files [file 41467_2020_17088_MOESM3_ESM.pdf]

**Description of Additional Supplementary Files**

File Name: Supplementary Movie 1
Description: Movie corresponding to Figure 1f (+NamptMO) showing a Nampt-depleted oocyte producing an enlarged PB. Time is shown as hh:mm following washout from IBMX.

File Name: Supplementary Movie 2
Description: Movie corresponding to Figure 2a (upper panels) showing a Mock-depleted oocyte treated with SiR-Tubulin (green, spindle) illustrating migration of the bipolar spindle to the cortex before anaphase-onset. Time is shown as hh:mm relative to GVBD.

File Name: Supplementary Movie 3
Description: Movie corresponding to Figure 2a (lower panels) showing a Nampt-depleted oocyte treated with SiRTubulin (green, spindle) illustrating migration of the bipolar s

File Name: Supplementary Movie 4
Description: Movie corresponding to Figure 4f showing an oocyte treated with SiR-Tubulin (green, spindle). Time is shown as hh:mm relative to anaphase-onset.

File Name: Supplementary Movie 5
Description: Movie corresponding to Figure 4g showing an oocyte treated with SiR-Tubulin (green, spindle). Time is shown as hh:mm relative to anaphase-onset.

File Name: Supplementary Movie 6
Description: Movie corresponding to Figure 5a showing a mock-depleted oocyte expressing H2B-RFP (red, chromosomes) and treated with SiR-Tubulin (green, spindle). Time is shown as hh:mm relative to anaphase-onset.
