# Supplementary material for: Nampt-mediated spindle sizing secures a post-anaphase increase in spindle speed required for extreme asymmetry
Source: Nat Commun. 2020 Jul 7;11:3393. doi: 10.1038/s41467-020-17088-6 (PMC7341875; doi:10.1038/s41467-020-17088-6)
Supplement: Supplementary file 11 — Reporting Summary [file 41467_2020_17088_MOESM11_ESM.pdf]

nature research

Corresponding author(s): Hayden Homer

Last updated by author(s): May 28, 2020

Reporting Summary

Nature Research wishes to improve the reproducibility of the work that we publish. This form provides structure for consistency and transparency in reporting. For further information on Nature Research policies, see Authors & Referees and the Editorial Policy Checklist .

Statistics

For all statistical analyses, confirm that the following items are present in the figure legend, table legend, main text, or Methods section.

n/a	Confirmed	
☐	☒	The exact sample size (*n*) for each experimental group/condition, given as a discrete number and unit of measurement
☐	☒	A statement on whether measurements were taken from distinct samples or whether the same sample was measured repeatedly
☐	☒	The statistical test(s) used AND whether they are one- or two-sided<br>*Only common tests should be described solely by name; describe more complex techniques in the Methods section.*
☒	☐	A description of all covariates tested
☒	☐	A description of any assumptions or corrections, such as tests of normality and adjustment for multiple comparisons
☐	☒	A full description of the statistical parameters including central tendency (e.g. means) or other basic estimates (e.g. regression coefficient) AND variation (e.g. standard deviation) or associated estimates of uncertainty (e.g. confidence intervals)
☐	☒	For null hypothesis testing, the test statistic (e.g. *F*, *t*, *r*) with confidence intervals, effect sizes, degrees of freedom and *P* value noted<br>*Give P values as exact values whenever suitable.*
☒	☐	For Bayesian analysis, information on the choice of priors and Markov chain Monte Carlo settings
☒	☐	For hierarchical and complex designs, identification of the appropriate level for tests and full reporting of outcomes
☐	☒	Estimates of effect sizes (e.g. Cohen's *d*, Pearson's *r*), indicating how they were calculated

Our web collection on statistics for biologists contains articles on many of the points above.

Software and code

Policy information about availability of computer code

Data collection	Leica Application Suite X 3.5.5.19976
Data analysis	LAS X Core 3.7.1, Imaris software 9.5.0, GraphPad Prism 8, Adobe Photoshop 19.1.3

For manuscripts utilizing custom algorithms or software that are central to the research but not yet described in published literature, software must be made available to editors/reviewers. We strongly encourage code deposition in a community repository (e.g. GitHub). See the Nature Research guidelines for submitting code & software for further information.

Data

Policy information about availability of data

All manuscripts must include a data availability statement. This statement should provide the following information, where applicable:

- Accession codes, unique identifiers, or web links for publicly available datasets
- A list of figures that have associated raw data
- A description of any restrictions on data availability

The data that support the findings of this study are available from the corresponding author upon reasonable request. The source data underlying Figs. 1-7 and Supplementary Figs. 1-5 are provided as a Source Data file.

Field-specific reporting

Please select the one below that is the best fit for your research. If you are not sure, read the appropriate sections before making your selection.

☒ Life sciences ☐ Behavioural & social sciences ☐ Ecological, evolutionary & environmental sciences

Life sciences study design

All studies must disclose on these points even when the disclosure is negative.

Sample size	For the measurements based on live-oocyte imaging and immunofluorescence, all experiments were performed at least 3 times and involved detailed measurements on a minimum of 10 oocytes from at least 3 different mice. Based on the effect sizes we observed, this number of oocytes was adequate for demonstrating statistically significant differences as we have found previously (Wei, Greaney, Zhou & Homer 2018, Nat. Commun.). For immunofluorescence studies, we ensured that oocytes at very similar maturation stages were used by restricting analyses to oocytes that underwent GVBD within 0.5 h of release from IBMX. Usually about 11% of oocytes undergo GVBD within 0.5 h.<br>For immunoblotting, we didn't predetermine the sample size. We tested each antibody to determine how many oocytes (using a minimum of 15 oocytes) were required to produce a clear band whose intensity could be reproducibly quantified. All experiments were performed at least 3 times and involved oocytes obtained from at least 3 mice.
Data exclusions	Throughout the paper, oocytes from an inbred BL6/CBAF1 strain were used. Females at 3-4 weeks were selected at random to be hormonally primed prior to being euthanased for obtaining oocytes. Only fully-grown cumulus-covered oocytes that underwent GVBD within 2 h of release from IBMX were included in experiments as is the standard for research with mouse oocytes. For experiments requiring measurements for bipolar spindles, we only included oocytes whose spindles remained in the same horizontal plane throughout the post-anaphase-onset period of migration. For experiments requiring detailed measurements for the spindles, protrusions and bulges at peri-anaphase, we only included oocytes whose spindles remained in the same horizontal plane, the same orientation and could be visualised concurrently with the emerging protrusion (membrane or cortex). This was important because any deviation of the spindle from the horizontal would impact the measurement value.
Replication	All experiments were performed at least 3 times and a minimum of 10 oocytes were used for the measurements. Usually variance among the samples was not big as the dots of the samples were clustered in the graph. Importantly, the phenotypes caused by Nampt-depletion were consistent and showed statistically significant differences from controls. We do note that in one case, the variability within the Nampt-depletion group was larger than the control group. In contrast to controls in which furrowing occurred roughly halfway along the spindle, following Nampt-depletion, membrane ingression occurred more randomly along the spindle length, typically in an off-centre position closer to the leading spindle pole (Fig. 5d). That's why the variability within the Nampt-depletion group was larger and it was one of our discoveries in the manuscript. Although the variability within the Nampt-depletion group was larger, the phenotype was very reproducible. So all attempts at replication were successful.
Randomization	For a typical experiment, 3 or more mice were hormonally primed ~44-46 h prior to euthanasia for obtaining ovaries. Oocytes from all ovaries were then pooled together and randomly allocated to different treatment groups.
Blinding	The investigators were not blinded. Using oocytes of differing growth sizes or that undergo GVBD at widely differing times could introduce an independent variable that could affect results. For consistency, we therefore selected only fully-grown oocytes and oocytes that underwent GVBD within 2 h since these are the most meiotically competent. Oocytes were then randomly allocated to either the treatment or control groups. From that point onwards, we needed to keep the two groups separate and identified by label at all stages since there was no other way of being certain that we were reporting on the correct group.

Reporting for specific materials, systems and methods

We require information from authors about some types of materials, experimental systems and methods used in many studies. Here, indicate whether each material, system or method listed is relevant to your study. If you are not sure if a list item applies to your research, read the appropriate section before selecting a response.

Materials & experimental systems

n/a	Involved in the study	
☐	☒	Antibodies
☒	☐	Eukaryotic cell lines
☒	☐	Palaeontology
☐	☒	Animals and other organisms
☒	☐	Human research participants
☒	☐	Clinical data

Methods

n/a	Involved in the study	
☒	☐	ChIP-seq
☒	☐	Flow cytometry
☒	☐	MRI-based neuroimaging

Antibodies

Antibodies used	rabbit anti-Nampt (AdipoGen-AG-25A-0028), rabbit anti-Acetylated-α-Tubulin (Cell Signaling Technology- 5335S), rabbit anti-Sirt2 (Sigma-Aldrich-S8447), mouse anti-Vinculin (Sigma- Aldrich-V9131), mouse anti-Actin (Millipore-MAB1501R) and rabbit anti-TOM20 (Santa Cruz-SC-11415), goat anti-mouse IgG (H L)-HRP conjugate (Bio-Rad, 170-6516), goat anti-rabbit IgG (H L)-HRP conjugate (Bio-Rad, 172-1019), Alexa Fluor 488 goat anti-mouse IgG (H+L) antibody (Thermo Fisher, A11001), Alexa Fluor 546 goat anti-rabbit IgG (H+L) antibody (Thermo Fisher, A11010)
Validation	1. rabbit anti-Nampt (AdipoGen-AG-25A-0028), Source/Host: Rabbit. Application: ELISA; Western Blot. Validation on the website:

Tested on recombinant proteins and/or target-protein transfected cell lines in ELISA, Western Blot and/or FACS. Specificity: mouse, weakly cross-reacts with human Nampt. Detects a band of ~54 kDa by Western blot. See website (https://adipogen.com/ag-25a-0028-anti-nampt-visfatin-pbef-mouse-pab.html/) for more details. For immunofluorescence, Nampt antibody was validated by identifying the fluorescent signals in oocytes, which decreased with morpholino-induced knockdown in our manuscript.

2. rabbit anti-Acetylated-α-Tubulin (Cell Signaling Technology-5335S), Source/Host: Rabbit. Application: Western Blot; immunofluorescence; flow cytometric analysis. Validation on the website: Western blot analysis of extracts from HeLa cells, untreated or Trichostatin A (TSA)-treated. The acetyl-specificity of the antibody was verified by blocking with an acetyl- or non-acetylpeptide. Confocal immunofluorescent analysis of rat testes. Confocal immunofluorescent analysis of HeLa cells, untreated or treated with Trichostatin A. Flow cytometric analysis of HeLa cells, untreated or treated with Trichostatin A (TSA). Reactivity: human, mouse, rat, monkey, zebrafish. Detects a band of ~52 kDa by Western blot. See website (https://www.cellsignal.com/products/primary-antibodies/acetyl-a-tubulin-lys40-d20g3-xp-rabbit-mab/5335?site-search type=Products&N=4294956287&Ntt=5335s&fromPage=plp&_requestid=4045268) for more details.

3. rabbit anti-Sirt2 (Sigma-Aldrich-S8447), Source/Host: Rabbit. Application: Western Blot; immunofluorescence; immunoprecipitation. Validation on the website: Whole extracts of mouse brain and rat brain were separated on SDS-PAGE and probed with this antibody. Fixed HeLa cells were stained with the antibody. This antibody was also used to immunoprecipitate Sirt2 from COS7 cells overexpressing human Sirt2. Reactivity: mouse, human, rat. Detects a band of ~37 kDa and 43 kDa by Western blot. See website (https://www.sigmaaldrich.com/catalog/product/sigma/s8447?lang=en&region=AU) for more details.

4. mouse anti-Vinculin (Sigma-Aldrich-V9131), Source/Host: Mouse. Application: Western Blot; immunofluorescence; immunohistochemistry. Validation on the website: Cell line lysates were separated on SDS-PAGE and probed this antibody for Western Blot. Several kinds of cells were labelled with anti-Vinculin for immunofluorescence. Reactivity: bovine, canine, mouse, rat, turkey, human, chicken, frog. Detects a band of ~116 kDa by Western blot. See website (https://www.sigmaaldrich.com/catalog/product/SIGMA/V9131?lang=en&region=AU) for more details.

5. mouse anti-Actin (Millipore-MAB1501R), Source/Host: Mouse. Application: ELISA; Immunocytochemistry; Immunohistochemistry; Immunohistochemistry (Paraffin); Western Blot. Validation on the website: Immunocytochemistry, confocal IF analysis of Hela, NIH/3T3 using this antibody. Western Blot, HEK293 cell lysate was resolved by electrophoresis, transferred to PVDF and probed with the antibody. Reactivity: all. Detects a band of ~43 kDa by Western blot. See website (https://www.merckmillipore.com/AU/en/product/Anti-Actin-Antibodyclone-C4,MM_NF-MAB1501R#overview) for more details.

6. rabbit anti-TOM20 (Santa Cruz-SC-11415), this antibody was used as validated previously for labelling mitochondria in mouse oocytes (Dalton & Carroll 2013 J Cell Sci).

Animals and other organisms

Policy information about studies involving animals; ARRIVE guidelines recommended for reporting animal research

Laboratory animals	Mouse, B6CBAF1, female, 3-4 weeks old. See "Methods" for details.
Wild animals	No wild animals were used in the study.
Field-collected samples	No field collected samples were used in the study.
Ethics oversight	All experiments presented are approved by the Animal Ethics Committee at the University of Queensland, approval number MED/UQCCR/475/15/NHMRC.

Note that full information on the approval of the study protocol must also be provided in the manuscript.
